# **Molecular diagnosis and anti-microbial resistance patterns among** ***Shigella*** **spp. isolated from patients with diarrhea**

**Published:** 2016

**Authors:** Hossein Hosseini Nave, Shahla Mansouri, Amin Sadeghi, Mohammad Moradi

**Affiliations:** *Department of Microbiology and Virology, School of Medicine, Kerman University of Medical Sciences, Kerman, IR Iran *

**Keywords:** Multiplex PCR, *Shigella flexneri*, *Shigella sonnei*, third-generation cephalosporins

## Abstract

**Aim:**

This study aims to determine the serogroup distribution and molecular diagnosis, as well as antimicrobial resistance profiles among *Shigella* spp. isolated from patients with diarrhea in Kerman, southeast of Iran.

**Background:**

*Shigella* species are frequent cause of bacterial dysentery worldwide. Previous studies have been reported that *S. sonnei* and *S. flexneri* are the most prevalent serogroups in various parts of Iran.

**Patients and methods:**

A total of 624 stool samples were randomly collected from patients with diarrhea from June 2013 to August 2014. Biochemical and serological characterizations were performed for identifying *Shigella *spp. In addition, the multiplex PCR assay was carried out for the detection and differentiation of three pathogenic *Shigella* spp. Antibiotic susceptibility testing was performed according to the Clinical Laboratory Standards Institute (CLSI) guidelines.

**Results:**

Fifty six (9%) *Shigella* strains were isolated from stool samples. The most common species were *S. flexneri* 31(55.4%), followed by *S* *.sonnei* 18(32.1%) and *S. boydii* 7(12.5%). *S. dysentery* was not detected in the present study. All the isolates that identified by serological test as *Shigella* spp. were confirmed by the multiplex PCR method. The highest rate of resistance was observed for ampicillin and trimethoprim-sulphamethoxazole antibiotics with 52(92.9%) resistant, followed by tetracycline 44(78.6%) and cefotaxime 33(58.9%). All *Shigella* isolates were susceptible to ciprofloxacin. A significant relationship was found between the *Shigella* species and cefotaxime resistance (*p*<0.05).

**Conclusion:**

*S. flexneri* was found as the most prevalent serogroup causing shigellosis. The high rate of resistance to third-generation cephalosporins limits the treatment options available for the management of shigellosis in Kerman, Iran.

## Introduction

 Shigellosis is an acute gastroenteritis infection and one of the most common causes of morbidity and mortality, especially in children with diarrhea in developing countries (1). Four species of *Shigella*, namely, *Shigella* *dysenteriae*, *S.* *flexneri*, *S. boydii*, and *S. sonnei* are responsible for shigelosis in humans (2). The most prevalent *Shigella* species in developed and developing countries are *S.* *sonnei* and *S. flexneri*, respectively (2). *S. flexneri* had been recognized as the mainspring of shigellosis in Tehran until 2003 (3, 4), then it was replaced by *S.* *sonnei* (2, 5). Nowadays, there are several methods for detection of *Shigella* species, including conventional culture and molecular techniques (6). Conventional methods have some limitations due to problems in processing and relying on the viable organisms (7). Moreover, these methods require several days to give reliable results (8). In various studies, molecular techniques have been used for detecting *Shigella *spp. (9-12). Multiplex PCR is one of the most popular techniques has been used due to rapidity and its capability to detect and differentiate *Shigella* spp. in a single reaction (7, 13).

Antibiotic treatment for shigellosis is usually recommended to reduce the severity of symptoms and potentially lethal complications and shorten the duration of the fecal excretion of the *Shigella* (14). However, due to the emergence of multi-drug resistant strains, empirical treatment with antibiotics such as sulfonamides, tetracycline, ampicillin and trimethoprim-sulphamethoxazole were no longer recommended (15). Previous studies have been indicated that, antimicrobial resistance patterns among *Shigella* spp. are varying between the countries and even within a country. Therefore, it is necessary to determine the local anti-microbial resistance pattern for selecting a proper antibiotic to treat shigellosis (1, 16). The present study aims to determine the serogroup distribution and molecular diagnosis, as well as antimicrobial resistance pattern among *Shigella* spp. isolated from patients with diarrhea in Kerman, southeast of Iran. 

## Patients and Methods

**Sample collection and** ***Shigella*** **identification**

A total of 624 stool samples were randomly collected since June 2013 to August 2014 from patients with diarrhea admitted to the hospitals of Afzalipour and Ayatollah Kashani in Kerman, Iran. The stools were immediately cultured on xylose lysine deoxycholate agar (XLD) and MacConkey agar media. Then, lactose-negative colonies were selected and identified as *Shigella* by standard biochemical methods (17) and were grouped serologically by slide agglutination with specific antisera (MAST Group LTD, Merseyside, UK). The isolates were kept in the TSB broth containing 30% glycerol at -70°C until testing.

**Table 1:** Sequences of primers used for the multiplex PCR

Reference	Species	Repeat size (bp)	Primer sequence	Primers
(7)	*S.boydii*	248	TCTGATGTCACTCTTTGCGAGT GAATCCGGTACCCGTAAGGT	B-F B-R
(7)	*S.sonnei*	503	AATGCCGTAAGGAATGCAAG CTTGAAGGAGATTCGCTGCT	SF SR
(13)	*S.flexneri*	537	TTTATGGCTTCTTTGTCGGC CTGCGTGATCCGACCATG	SflexDF1 SflexDR1

**Identification of the** ***Shigella*** **species through PCR**

For PCR amplification, DNA template was obtained as previously described by Ranjbar *et al*. (18). *Shigella *species were identified using specific primers of each species. The sequences of the primers are shown in Table 1. PCR reactions were performed in a final volume of 20 µl using the 3µl DNA solution, 10 µl Master mix (Amplicon, Brighton, UK), 10 pmol primer and water. Amplification was carried out in the thermo cycler (Biometra-T gradient, Biometra GmbH, Gottingen, Germany). The PCR conditions were as follows: initial denaturation at 95°C for 5 min, followed by 30 cycles, including denaturation at 95°C for 45 seconds, annealing at 56 for 35 seconds and extension at 72°C for 45 seconds and a single final extension at 72°C for 5 minutes.

**Table 2:** Antibiotic resistance patterns of the *Shigella *spp

Total No. (%)	*S. boydii* No. (%)	*S. sonnei* No. (%)	*S. flexneri* No. (%)	Antibiotic
52(92.9)	7(100)	18(100)	27(87.1)	Trimethoprim-sulphamethoxazole
52(92.9)	6(85.7)	17(94.4)	29(93.5)	Ampicillin
44(78.6)	5(71.4)	17(94.4)	22(71)	Tetracycline
23(41.1)	2(28.6)	1(5.6)	20(64.5)	Chloramphenicol
9(16.1)	0(0)	8(44.4)	1(3.2)	Gentamicin
27(48.2)	3(42.9)	13(72.2)	11(35.5)	Ceftriaxone
33(58.9)	4(57.1)	16(88.9)	13(41.9)	Cefotaxime
19(33.9)	2(28.6)	6(33.3)	11(35.5)	Aztreonam
12(21.4)	1(14.3)	3(16.7)	8(25.8)	Nalidixic-acid
3(5.4)	0(0)	0(0)	3(9.7)	Ofloxacin (5μg)
0(0)	0(0)	0(0)	0(0)	Ciprofloxacin (5μg)

**Figure 1:**
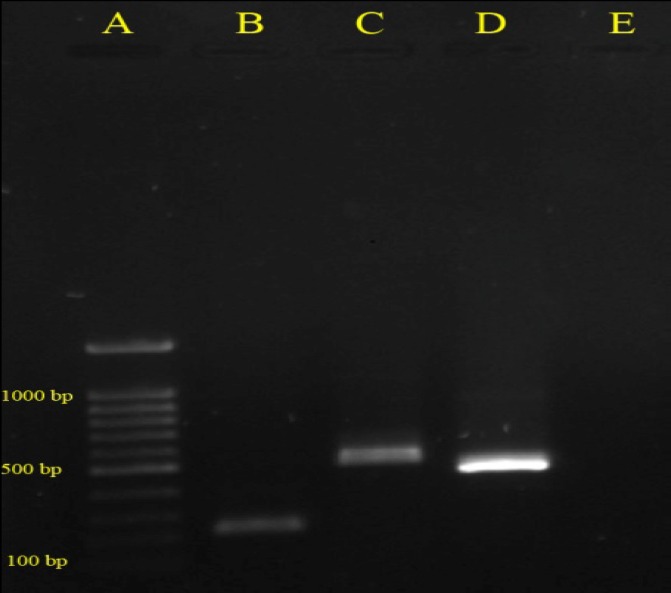
Detection of specific *Shigella* species genes by multiplex PCR, A: Marker- 100 bp, B: *S. boydii*- 248 bp, C: *S. flexneri*- 537 bp, D: *S. sonnei*- 503 bp, E: Negative Control

**Antimicrobial susceptibility testing**

Antimicrobial susceptibility testing for 11 antibiotics, including: chloramphenicol (30μg), ampicillin (10μg), trimethoprim-sulphamethoxazole (1.25/23.75μg), azteronam (30μg), gentamicin (10μg), cefotaxime (30μg), ceftriaxone (30μg), tetracycline (30μg), nalidixic acid (30μg), ofloxacin (5μg) and ciprofloxacin (5μg) was determined by disk diffusion method according to the Clinical Laboratory Standards Institute guidelines (CLSI) (19). *E. coli* ATCC 25922 was used as the anti-biogram control.

**Statistical analysis**

The SPSS (Statistical Product and Service Solutions, version 20.0) (SPSS Inc., Chicago, IL, USA) was used for the analysis of data. We used the chi-square test to compare proportions and Fisher’s exact test when appropriate. A value of p<0.05 was considered statistically significant. 

## Results

Fifty six (9%) *Shigella* strains were isolated from 624 stool samples. *S. flexneri* was the most frequently isolated serogroup 31(55.4%), followed by *S. sonnei* 18(32.1%), and *S. boydii *7(12.5%). *S. dysentery *was not detected in the present study. All isolates that identified by serological test as *Shigella* spp. were confirmed by the multiplex PCR method. Multiplex PCR was successfully developed and optimized for rapid, accurate and simultaneous detection of *Shigella* species (Fig.1). As shown in Table 2, the highest resistance rate was observed for ampicillin and trimethoprim-sulphamethoxazole 52(92.9%), followed by tetracycline 44(78.6%) and nalidixic acid 12(21.4%). Notably, *Shigella* spp. were also resistant to the third generation cephalosporins, so that 27 (48.2%) and 33 (58.9%) of isolates were resistant to ceftriaxone and cefotaxime, respectively. Despite observed resistance towards the third generation cephalosporins in all serogroups, *S.* *sonnei* strains were highly resistant to these antibiotics, so that 16(88.9%) of its isolates were cefotaxime resistant and 13(72.2%) isolates were ceftriaxone resistant. A significant relationship was found between the *shigella* species and cefotaxime resistance (*p*<0.05). Although resistance to quinolones and chloramphenicol was mainly found in *S. flexneri* strains, overall analysis showed higher resistance to antibiotics in the *S. sonnei* more than other species (Table 2). All *Shigella* isolates were susceptible to the ciprofloxacin. 

## Discussion

The present study investigated the prevalence of diarrhea caused by *Shigella* isolates and their antibiotic resistance in Kerman, southeast of Iran. In Tehran, Iran, *S. flexneri* was the predominant serogroup up to 2003 (3, 4). But recent studies indicated that it was replaced with *S. sonnei* (2, 5). In a study in Shiraz, Iran, *S.* *sonnei* was found to be the main cause of shigellosis (74.39%) (20). In the present study, however, *S. flexneri* was the most common serogroup causing shigellosis in Kerman. The results of this study are consistent with several other studies in different parts of Iran (21, 22). Differences in the dominant serogroup in Tehran and Shiraz compared to this study may be related to the higher levels of hygiene and development in those two cities (3).

Due to the speed, sensitivity and specificity, molecular methods have offered advantages over the conventional methods. However, some molecular techniques used to identify and differentiate the *Shigella* are relatively expensive, difficult to perform, and requiring special equipment. Some of these molecular techniques include: PCR-ELISA (11), seminested PCR (12), PCR-RFLP (9) and PCR-Nonradioactive labeling (10). Without these constraints, multiplex PCR method can serve as the valuable detective tool for diagnosis of *Shigella* species. Several previous studies have used multiplex PCR for rapid and simultaneous detection of *Shigella* species (7, 13). But it should be considered that molecular techniques complement rather than replace conventional methods.

The emergence of multidrug resistant (MDR) *Shigella* isolates has complicated the selection of empirical antibiotics for treatment of shigellosis (1). As many previous studies in Iran and other countries, high rate of resistance to ampicillin, trimethoprim-sulphamethoxazole and tetracycline was observed (3, 21-24). Due to the high resistance to these antibiotics, WHO suggested the ciprofloxacin as a drug of choice for the treatment of shigellosis in 1990 (14). However, the third generation cephalosporins are used as the alternative treatment of shigellosis because of the concerns about the side effects of ciprofloxacin in children and an increased resistance to it (25). In the present study, resistance to ciprofloxacin was not found. Therefore, it can be used as the treatment of choice in patients with shigellosis in this area. However, resistance to third-generation cephalosporins is really concerning because of their high importance in the shigellosis treatment especially in children. Despite previous reports on the resistance to third-generation cephalosporins in various parts of the world and Iran, This resistance rate is surprisingly high, and the findings of the current study do not support the previous studies (3, 5, 21-24). This result may be due to excessive using of beta-lactam antibiotics in treatment of infections, resulting in selecting the resistant strains and propagation of beta-lactamase genes in the normal intestinal flora. Reports indicate the easy transferring of the resistance genes from the commensal members of the Enterobacteriaceae family to the enteropathogens (23, 26). In consistent with our results, Farshad, *et al.* in Shiraz, Iran showed higher resistance rates to antibiotics in the *S. sonnei* strains than other serogroups (20). High resistance of the *S. sonnei* strains to the antibiotics of beta-lactam families and gentamicin will pose challenges in the treatment of infections caused by this serogroup.

In conclusion, *S. flexneri* was recognized as the dominant serogroup causing shigellosis in Kerman, Iran. The results showed that multiplex PCR can be used for identification of *Shigella* species with high speed and appropriate sensitivity and specificity. Empirical treatment for shigellosis entails the knowledge of the antimicrobial resistance pattern of local *Shigella* strains. Despite the high sensitivity to ciprofloxacin, the *Shigella* isolates showed resistance to the antibiotics like Trimethoprim-sulphamethoxazole and ampicillin in the present study. The high rate of resistance to third-generation cephalosporins limits the treatment options available for the management of shigellosis in Kerman, Iran. Continuous monitoring of antimicrobial resistance is necessary for appropriate antibiotic treatment of Shigellosis.
